# TGF-β Pathway in Salivary Gland Fibrosis

**DOI:** 10.3390/ijms21239138

**Published:** 2020-11-30

**Authors:** Xianglan Zhang, Jun Seop Yun, Dawool Han, Jong In Yook, Hyun Sil Kim, Eunae Sandra Cho

**Affiliations:** 1 Department of Pathology, Yanbian University Hospital, Yanji, Jilin 133000, China; zhangxianglan@yuhs.ac; 2 Oral Cancer Research Institute, Yonsei University College of Dentistry, Seoul 03722, Korea; yjs8714@yuhs.ac (J.S.Y.); ipodvideo@yuhs.ac (D.H.); jiyook@yuhs.ac (J.I.Y.); 3 Department of Oral Pathology, Yonsei University College of Dentistry, Seoul 03722, Korea; 4 BK21 FOUR Project, Yonsei University College of Dentistry, Seoul 03722, Korea

**Keywords:** TGF-β, BMP, salivary gland, fibrosis, sialadenitis, Sjögren’s syndrome, drug delivery

## Abstract

Fibrosis is presented in various physiologic and pathologic conditions of the salivary gland. Transforming growth factor beta (TGF-β) pathway has a pivotal role in the pathogenesis of fibrosis in several organs, including the salivary glands. Among the TGF-β superfamily members, TGF-β1 and 2 are pro-fibrotic ligands, whereas TGF-β3 and some bone morphogenetic proteins (BMPs) are anti-fibrotic ligands. TGF-β1 is thought to be associated with the pro-fibrotic pathogenesis of sialadenitis, post-radiation salivary gland dysfunction, and Sjögren’s syndrome. Potential therapeutic strategies that target multiple levels in the TGF-β pathway are under preclinical and clinical research for fibrosis. Despite the anti-fibrotic effect of BMPs, their in vivo delivery poses a challenge in terms of adequate clinical efficacy. In this article, we will review the relevance of TGF-β signaling in salivary gland fibrosis and advances of potential therapeutic options in the field.

## 1. Introduction

The transforming growth factor beta (TGF-β) signaling pathway is known for its pivotal role in human health and disease. The TGF-β superfamily is ubiquitous in physiologic tissue, although the expression of ligands and receptors are tissue-, cell- and condition-specific [1,2]. TGF-β family members regulate tissue morphogenesis, cellular differentiation, proliferation, migration, angiogenesis, immune response and extracellular matrix (ECM) deposition, being critical for tissue homeostasis [3,4,5,6,7]. Altered TGF-β signaling leads to a wide spectrum of diseases, including fibrosis, autoimmune disease, inflammatory diseases, and cancer [7,8,9]. Salivary glands, exocrine glands that secrete saliva into the oral cavity, are among the many organs in which TGF-β signaling has significance in both physiologic homeostasis and disease.

Salivary gland dysfunction is caused by aging, inflammation, infection, physical compression (by tumor or sialolith), medications, radiation therapy for head and neck cancer, and autoimmune disease [10,11]. Saliva is essential to maintaining a healthy oral environment as it protects the teeth and oral mucosa through its antimicrobial, lubricant, and buffering effects. Altered salivary flow or composition may lead to xerostomia, dental caries and erosion, stomatitis, mucosal pain, difficulties in food intake and debris clearing, taste disturbance, speech problems and depression [10]. Persistently reduced salivary functions are usually caused by acinar atrophy, interstitial fibrosis, or adipose tissue replacement. In this article, we will focus on the role of TGF-β signaling in fibrosis of salivary glands and review recent developments relevant to potential therapeutic strategies in the field.

## 2. Overview of the TGF-β Signaling Pathway 

### 2.1. TGF-β Family Members and Downstream Signaling

The TGF-β family comprises a wide group of mainly extracellular ligands, TGF-βs, bone morphogenetic proteins (BMPs), activins, growth and differentiation factors, nodals, and Müllerian inhibitory substance. The family name is due to the characteristics of the first member discovered [5]. Given the wide spectrum of TGF-β family members, we will focus only on those few representative members relevant to salivary gland tissue homeostasis and diseases, namely, three of the five mammalian isoforms (TGF-β1, 2 and 3) and a few notable bone morphogenetic proteins (BMPs) [5]. Of the five TGF-βs, which have a highly homologous peptide sequence, only the three primary TGF-βs are expressed in humans, TGF-β4 and 5 having been noted only in chicken and frog, respectively [12,13]. The original list of six types of BMPs in the TGF-β family (BMP2 to 7) has, to date, expanded to more than twenty types, some having both secreted growth factor and nuclear localized forms [14]. TGF-β1 and BMP9 procomplexes share only a minimal portion of peptide sequence and structural homologies, indicating the diversity among TGF-β family members [13,15,16]. 

**Figure 1:**
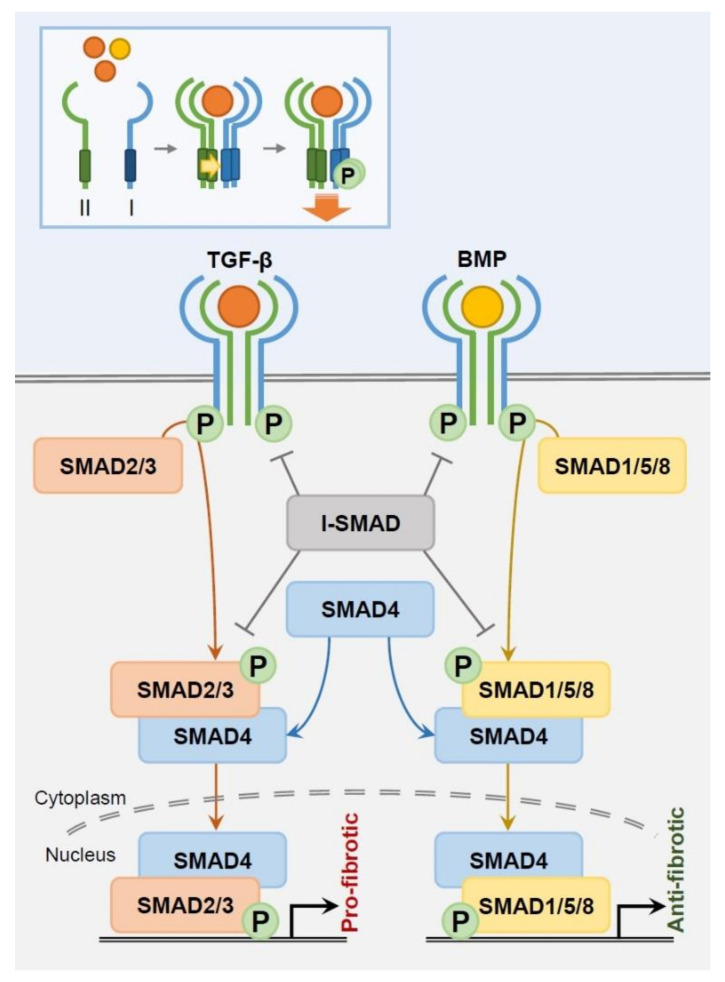
Schematic image of TGF-β/SMAD and BMP/SMAD signaling pathway in fibrosis (upper left box, signal activation via TGF-β receptor and ligand attachment; orange and yellow circles/arrows, TGF-β ligands/signaling and BMP ligands/signaling, respectively). Abbreviations: TGF-β, transforming growth factor beta; BMP, bone morphogenetic protein; P, phosphorylation; I-SMAD, inhibitory SMAD; I, TGFβR1; II, TGFβR2.

Despite this diversity of subtypes, TGF-β family members share a range of recognized surface receptors (TGFβR) and downstream signaling pathways (Figure 1). In mammals, TGF-β family members bind to a specific combination of seven type I (TGFβR1), five type II (TGFβR2), and one type III (TGFβR3) receptors, thus enabling selective signaling [17,18]. The TGF-β family member ligands correspond to transmembrane receptors TGFβR1 and TGFβR2, which have a dual-specific cytoplasmic kinase domain of both serine/threonine kinase and tyrosine kinase activity [19]. Generally designated as serine/threonine kinase in literature, the receptors form a heterotetrameric complex composed of two of each type I and type II receptor for activation. This tightly regulated, specific signaling process is supported through a 100-fold higher ligand/receptor affinity and a 10-fold lower receptor density compared to other growth factor ligands/receptor tyrosine kinase binding [20,21,22]. Type I receptors, also called activin receptor-like kinases (ALKs), are phosphorylated by Type II receptors upon ligand attachment and henceforth enter the canonical SMAD pathway. Meanwhile, TGFβR3 (β-glycan), not a receptor kinase, functions as a co-receptor that presents ligands to the two TGF-β kinase receptors rather than passing down direct signaling [23].

Highly conserved SMAD proteins are tightly coordinated to regulate TGF-β-derived transcription (Figure 1). SMAD2 and 3 are TGFβR -regulated (R)-SMADs, while SMAD1, 5 and 8 are BMP R-SMADs [24]. During intracellular signaling, TGF-β and BMP R-SMADs each join with common partner (Co)-SMAD4 in a heteromeric complex to translocate into the nucleus as transcription factors. Inhibitory (I)-SMADs SMAD6 and 7 negatively interact with type I receptors and R-SMADs to suppress intracellular SMAD signaling. I-SMAD6 predominantly inhibits BMP type I receptors ALK-3 and 6 and is an antagonist competitor of SMAD4, creating an inactive SMAD1/SMAD6 complex instead of the active SMAD1/SMAD4 complex [25,26]. This blocks BMP signaling despite BMP receptor-mediated SMAD1 phosphorylation [26]. SMAD6 can nevertheless disrupt SMAD2, but not SMAD3, phosphorylation. SMAD6 receptor affinity is weaker in TGF-β type receptors than in BMP type receptors, limiting its relevance to TGF-β signaling [25,27]. In contrast, BMP7 is ubiquitously interactive with both TGF-β and BMP pathways. SMAD7 suppresses TGF-β and BMP type I receptor-mediated phosphorylation, as well as phosphorylation of SMAD2 and 3, inhibiting further downstream signaling [28,29,30]. Importantly, TGF-β1 itself drives immediate SMAD7 mRNA transcription, indicating that TGF-β signal regulation significantly relies on a SMAD7-mediated feedback loop [30]. 

Alternatively, TGF-β family members can deliver signals via non-SMAD pathways, such as ERK (extracellular signal-regulated kinase), JNK (c-Jun N-terminal kinase), p38 MAPK (mitogen-activated protein kinase), PI3K (phosphoinositide 3-kinase), Rho family of GTPases and IKK (IκB kinase)/NF-κB (nuclear factor kappa-light-chain-enhancer of activated B cells) [31,32,33,34,35,36]. These non-SMAD pathways can signal as independent streams or may crosstalk with SMAD pathways at multiple levels. Therefore, targeting TGF-β family downstream factors for therapeutic purposes is appealing, yet tricky, due to these multiple pathway interactions as well as their widespread distribution in physiologic tissue. 

### 2.2. TGF-β Latency and Activation

Ligand/receptor binding is not the only requirement for TGF-β signal activation. The unique structure of TGF-β isoforms organizes a non-active state upon secretion. Thus, TGF-β activity level is not absolutely equivalent to TGF-β synthesis or expression signature. TGF-β family members are synthesized with prodomains cleaved by proteases before secretion [37,38,39,40]. In some family members, the mature peptide remains attached to the propeptides by additional noncovalent bonds despite prior prodomain cleavage [41]. TGF-β1, 2, 3, and BMP2, 4, 7, 10 present this latency prodomain segment although the overall structure differs among subtypes [42,43]. 

**Figure 2:**
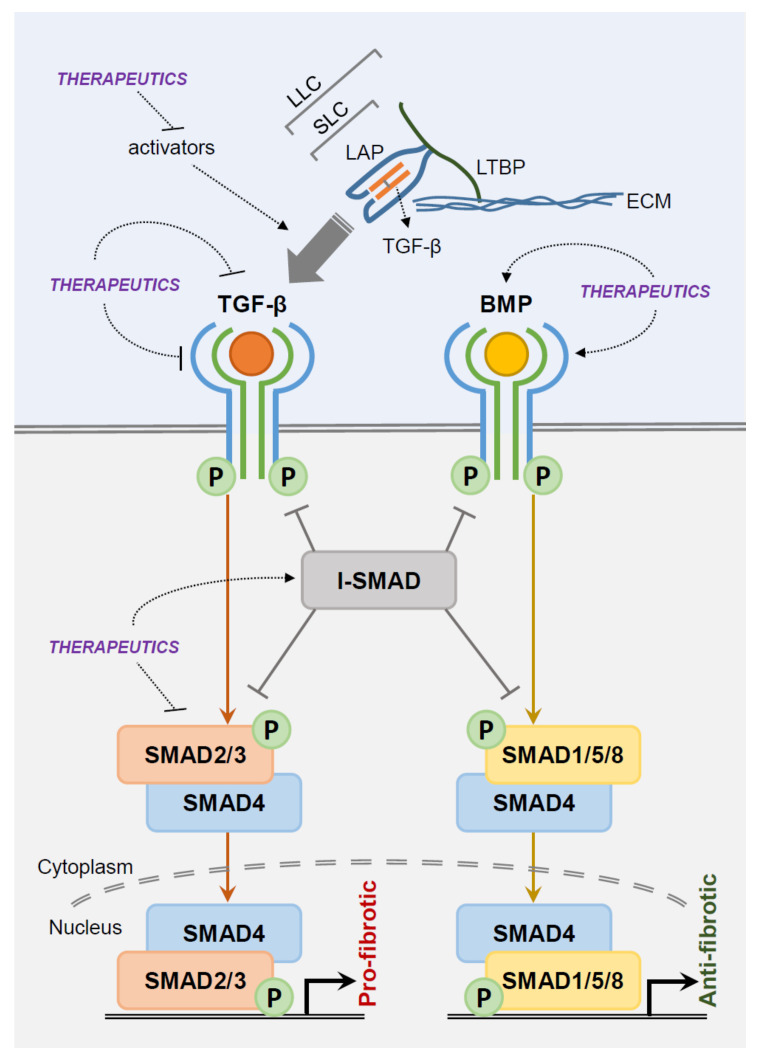
Potential therapeutic target sites in the TGF-β/BMP signaling pathway. (Orange and yellow circles/arrows, TGF-β ligands/signaling and BMP ligands/signaling, respectively). Abbreviations: TGF-β, transforming growth factor beta; BMP, bone morphogenetic protein; P, phosphorylation; I-SMAD, inhibitory SMAD; LAP, latency-associated proteins; LTBP, latent TGF-β binding protein; ECM, extracellular matrix; SLC, small latent complex; LLC, large latent complex.

The dynamics and structures of latent ligands in TGF-β isoforms have been well described (Figure 2). The mature TGF-β dimers are surrounded by dimeric propeptides called latency-associated proteins (LAPs), creating a small latent complex (SLC). Isolation of the mature peptide and LAPs are essential for TGF-β activation. LAPs maintain latency by either direct interference of the mature peptide domain/receptor binding or alteration of the mature peptide structure [42]. The SLC is linked with latent TGF-β binding proteins (LTBPs) by disulfide bonds to form a large latent complex which, in turn, covalently binds to the ECM. LTBPs are not merely linkage proteins which maintain TGF-β latency, they arrange the ECM as a growth factor storage platform and are a necessary component in TGF-β activation by αvβ6 integrin [44,45,46]. These features contribute to TGF-β concentration in the ECM.

## 3. TGF-β Signaling in Fibrosis 

TGF-β signaling is essential to wound healing, the ultimate goal of which is to regain original tissue composition and function. In most cases, the physical condition of the wound, replicative capacity of the injured cells, tissue microenvironment, infection status, and systemic health status may interfere with full regeneration, resulting in repair instead. Repair is established by the termination of inflammation and substitution of the injured tissue through fibrosis, composed by excessive ECM accumulation and remodeling. The inflammatory wound environment has the potential to activate latent forms of TGF-β1 concentrated within the ECM, inducing a pro-fibrotic response [47,48]. Fibrosis is a practical alternative to regeneration, but uncontrolled fibrosis results in scarring and organ dysfunction that sometimes may be fatal.

TGF-β isoforms, particularly TGF-β1, are critical for fibrosis pathogenesis, as proven by preclinical evidence: (1) Exogenous TGF-β inserted in healthy animal models presented increased fibrosis, (2) TGF-β overexpressed in transgenic models exhibited prominent, often diffuse fibrosis, and (3) TGF-β inhibition reduced experimental fibrosis [49,50,51,52,53,54,55]. TGF-β promotes fibrosis by increased ECM synthesis and preservation, fibroblast activation, myofibroblast phenotype acquisition, epithelial–mesenchymal transition (EMT) and endothelial-mesenchymal transition (EndMT) [4,49,56,57,58]. 

TGF-β1 is well described for its myofibroblast and fibroblast regulation during fibrosis. TGF-β1 induced fibroblast activation resulting in myofibroblast differentiation has been reported as an important source of collagen, glycoproteins, proteoglycans, and matrix metallopeptidases (MMPs) in wound healing and fibrosis [59,60]. Fibrosis-associated myofibroblasts are assumed to have originated from epithelial cells, endothelial cells, and fibroblasts via the canonical SMAD pathway [4,57,61]. The obtained myofibroblasts are then lost by apoptosis during transition from granulation tissue to a localized scar in wound healing, although they are persistent in progressive fibrotic diseases [62,63]. Myofibroblasts have specific intracellular stress fibers and contractile function compared to fibroblasts, and α-smooth muscle actin (αSMA) expression increases with differentiation [64,65,66]. Contractile smooth muscle gene expressions are induced in fibroblasts via TGF-β1-dependent signaling and ECM molecule interactions [65,67,68]. This contractile phenotype in turn can further activate latent TGF-β1, possibly causing a positive feedback loop for fibrosis [65,69,70]. Therefore, myofibroblasts have been most commonly identified in vitro and in vivo through αSMA expression. Whether αSMA is a specific marker for myofibroblast is a controversial issue that is further discussed later in this article. 

The myofibroblast phenotype has been generally described as the ‘classic’ source of fibrotic proteins in the previous literature, whereas recent updates have suggested a more diverse phenotypic spectrum of fibroblastic cells in fibrosis. Stromal investigations of mouse and human fibrotic tissues revealed a prominent heterogeneous population of fibroblasts that had a ‘non-αSMA’ phenotype despite of collagen production [71,72,73]. The recent enablement of multiple marker expression at a single cell level by flow cytometry in human samples has given us a new level of insights on the heterogeneity and plasticity of fibrosis-associated molecules and cells [72,74,75]. Collagen Triple Helix Repeat-Containing-1 (CTHRC1) is of particular interest in TGF-β1 regulated fibrosis with conflicting interpretations among preclinical studies. Several studies have proposed an anti-fibrotic effect of CTHRC1 in experimental pulmonary, cholestatic, and cutaneous fibrosis via reciprocal regulation with TGF-β signaling [76,77,78]. CTHRC1 transcription was shown to be induced through TGF-β1 activation via direct interaction of phosphorylated SMAD3 at the promoter [77]. In turn, the CTHRC1 protein inhibited SMAD3 phosphorylation by degradation in fibroblasts and smooth muscle cells, forming a negative feedback loop in TGF-β, but not BMP downstream SMAD signaling [77,79]. In contrast, CTHRC1-null transgenic mice presented suppressed SMAD3 phosphorylation and alleviated hepatic fibrosis [80]. Interestingly, a recent single cell RNA sequencing study on human normal and fibrotic lung tissue presented that CTHRC1 was predominantly expressed in cells from pathologic fibrosis tissue and correlated with collagen gene expression [72]. This rather indicated a pro-fibrotic role of CTHRC1 in human pulmonary fibrosis. Only a fraction of COL1A1 and CTHRC1 positive cells co-expressed with ACTA2 (gene of αSMA protein) in human idiopathic pulmonary fibrosis and scleroderma, implying the clinical existence of other ‘non-classic’ fibroblast phenotypes involved in fibrosis [72]. Moreover, a single cell analysis of murine pulmonary fibrosis suggested the existence of low αSMA-expressing matrix fibroblasts (which tend to expand during fibrosis) might have been improperly distinguished as myofibroblasts in previous studies [75]. Due to these advances in technology, we are now just beginning to gain details on how TGF-β signaling regulates heterogeneous cellular subtypes in fibrosis [74]. 

Less described than TGF-β1, TGF-β2 is a known fibrosis inducer as well. One study has proposed that TGF-β2 may work along with TGF-β1 in a synergetic manner rather than as an independent factor [81,82,83]. Unlike TGF-β1 and 2, TGF-β3 promotes wound healing without scarring, indicating an anti-fibrotic effect [84,85,86]. TGF-β3 suppressed pro-fibrotic gene transcription, myofibroblast differentiation, and ECM synthesis/remodeling, while inducing re-epithelialization during wound healing [85,86]. Taking these together, we see that TGF-β isoforms each have specific effects on fibrosis. TGF-β1 is the key regulator of fibrosis, whereas TGF-β3 acts as its antagonist. 

The majority of the current preclinical research recognizes BMPs to be anti-fibrotic, the most clearly anti-fibrotic being BMP7. BMP7 is expressed in specific types of normal mature tissue, representatively the kidney [87,88]. BMP7 mRNA expression is seen in mature murine salivary gland tissue [89]. In contrast to normal tissue of rats, BMP7 and its receptors disappeared in interstitial fibrosis of diabetic nephropathy [90]. Loss of ALK-3, the receptor of BMP7, increased TGF-β1/SMAD axis-derived fibrosis, indicating the critical role of BMP7 and its downstream signaling in anti-fibrotic activity [91]. Additionally, BMP2, 4 and 6 bind to ALK-3, and BMP2 and 6 have been reported to be protective of fibrosis [92,93,94,95,96,97]. Meanwhile, there are conflicting data on the regulation of fibrosis by BMP4 [98,99,100,101]. BMPs can attenuate fibrosis through SMAD7 activity or competition with pro-fibrotic TGF-β for SMAD4 to reduce myofibroblast differentiation (BMP7), EMT (BMP2 and 7) and ECM synthesis/remodeling (BMP4 and 7) [98,102,103,104,105,106]. 

## 4. TGF-β Signaling in Salivary Gland Fibrosis 

Indeed, aberrant TGF-β signaling has been recognized in a wide spectrum of fibrotic diseases, not limited to systemic sclerosis [107], keloid [108], pulmonary fibrosis [109], hepatic fibrosis [110], renal fibrosis [111], cardiac fibrosis [112], myopathies [113], and salivary gland fibrosis [114]. Salivary gland fibrosis can be seen in chronic inflammation, aging, infections, ductal obstruction, physical trauma, irradiation, and autoimmune disease [10,11,115]. 

### 4.1. Sialadenitis

**Figure 3:**
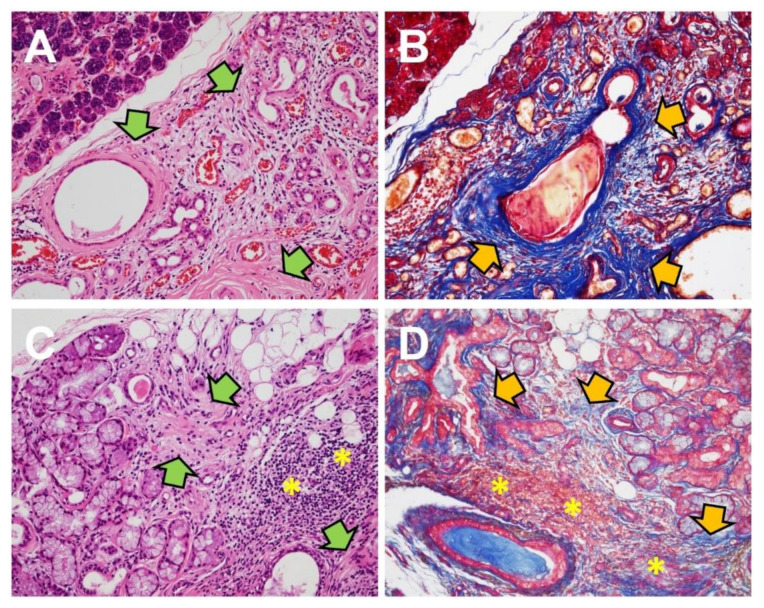
Fibrosis in salivary gland diseases. Under hematoxylin and eosin stain (left) and Masson’s trichrome stain (right), fibrosis is presented as dense pink collagen bundles (green arrows) and dense blue collagen bundles (yellow arrows), respectively. (**A**,**B**). Fibrosis in sialadenitis of the submandibular gland (×200). (**C**,**D**). Fibrosis adjacent to lymphocytic aggregation (asterisks) in labial minor salivary glands diagnosed as Sjögren syndrome (×200).

Although human specimens for acute salivary gland injury and wound healing are usually not available for gross or pathological examination, animal models give us a partial view of the process. Chronic sialadenitis is composed of chronic inflammatory cells, acinar atrophy, and prominent fibrosis (Figure 3A,B). Clinical samples of human chronic obstructive sialadenitis have been found to express TGF-β [116,117]. Mice models with intentional ductal ligation in salivary glands showed robust expression of TGF-β1, 3 and TGFβR1, but not TGF-β2 [118,119]. The use of exogenous TGFβR1 inhibitor in the mice model reduced fibrotic markers in ligation-induced salivary gland injury [119]. These data are consistent with TGF-β signaling in models of bile duct ligation-induced liver fibrosis [120,121]. The separate roles of TGF-β signaling in the inflammatory portion and fibrotic portion are detailed below under “Sjögren’s syndrome”. 

### 4.2. Post-Radiation Induced Salivary Gland Fibrosis

TGF-β signaling has been well established in post-radiation induced organ fibrosis, including salivary gland fibrosis in irradiated head and neck cancer patients [122,123]. TGF-β protein expression increased about 10-fold in salivary gland specimens of patients with radiation-induced salivary dysfunction [124]. In irradiated mouse models, TGF-β1 expression increased after irradiation, and again decreased after hyperbaric oxygen therapy [125]. Furthermore, other pro-fibrotic genes followed a similar pattern, such as TGF-β1 after hyperbaric oxygen therapy, despite the lack of actual fibrotic histology in the specimens. 

### 4.3. Sjögren’s Syndrome

Sjögren’s syndrome (SS) is a significant autoimmune disease with primary symptoms in the salivary and lacrimal glands, typically dryness. SS can be accompanied by other autoimmune diseases or connective diseases, such as rheumatoid arthritis or systemic lupus erythematosus [126]. Circulating autoantibodies or histological confirmation is required for diagnosis in SS [127]. The typical histology of SS in the salivary glands is periductal lymphocytic aggregations with acinar atrophy, which may be accompanied by interstitial fibrosis (Figure 3C,D) [115]. TGF-β signaling has been analyzed separately in the inflammatory and fibrotic portion of SS in the previous literature. 

The critical role of TGF-β signaling in immune regulation has been verified in genetically modified mice models. Homozygous TGF-β1 deletion in mice reached full development, but was rapidly followed with fatal, early postnatal inflammatory infiltrations, wasting, and damage in multiple organs [128,129]. Salivary glands were a distinct target in TGF-β1-deficient mice models with histological resemblance to SS. The inflammatory cell composition in these models were specific to its infiltrated type of organ, and periductal lymphocytic infiltrations were predominant in salivary glands [128]. A T cell specific-SMAD4 deletion mice model presented higher lymphocytic infiltration at the exocrine glands, attenuated saliva/tear synthesis, and increased serum autoantibodies [130]. Serum autoantibodies developed in the genetic mice models targeted autoantigens SSA/Ro, SSB/La, dsDNA, ssDNA, and Sm, which were clinically relevant with real-life autoimmune patients [130,131,132,133]. TGF-β genetic mice models have implied the protective effect of TGF-β1 signaling in autoimmune disease development. 

Despite the rich phenotypic information we have gained from genetic mice models, the role of TGF-β in actual autoimmune diseases should not be simplified, as seen in the models. Current studies have described the double-faced functions of TGF-β in peripheral T lymphocytes. TGF-β1 promotes immunosuppressive activity by inhibition of effector T cell differentiation [134]. Furthermore, TGF-β1 can induce regulatory T cell differentiation by interleukin (IL)-2 mediated FOXP3 expression [135,136,137]. Meanwhile, TGF-β with IL-6 can activate pro-inflammatory responses through T helper (Th) 17 cell differentiation [138]. TGF-β-mediated Th17 cell differentiation is tightly coordinated with other multiple cytokines and is activated under certain conditions, such as the presence of T cell-made TGF- β1 [134,139,140]. The pathogenesis of SS is suspected as loss of immune tolerance and increased inflammatory factors, but the details require further research [141]. Whether TGF-β has a protective effect or pathogenic drive in SS needs in-depth investigations considering the content-dependent pleiotropic ability of TGF-β during immune regulation. 

Expression of BMP6, a TGF-β1 antagonist, was locally increased in minor salivary glands in SS patients and develops salivary dysfunction in mice [142,143,144]. Although the overexpression of BMP6 creates SS-like phenotypes in animal models, the underlying mechanism is not clear [143]. Xu et al. assumed that BMP6 may indirectly induce immune cell infiltration via mesenchymal stem cell function impairment [145]. Other studies suggested that BMP6-induced salivary gland dysfunction had a weak link with immune cell regulation and was rather associated with the loss of glandular cell water permeability or ECM modulation [142,143,144]. 

Fibrosis in minor salivary gland biopsies of SS patients is generally located at the periphery of immune infiltration at a higher degree than non-SS patients [146]. The degree of fibrosis may increase along with disease progression and immune focus score [115,147]. The fibrotic portion of SS is mediated by TGF-β signaling consistent with other fibrotic diseases. Chronic lymphocytic inflammation is presumed to stimulate TGF-β1-mediated EMT, which in turn can induce fibrosis adjacent to the lymphocytic aggregation, although this has only been confirmed in vitro [115,148,149]. Animal models have implied that TGF-β1 signaling seems to be more strongly associated with late stage fibrosis than early stage inflammation in autoimmune diseases [147]. In addition to SS, TGF-β is associated with fibrotic sclerosis in another autoimmune disease involving the salivary gland, IgG4-related disease, although the specific related isoform has not been determined [150,151]. We can refer from a study of experimental lupus that the role of TGF-β signaling in autoimmune diseases is dependent on the type of cell, histological location, and course of disease [147]. To unravel the complex role of TGF-β signaling in SS pathogenesis, TGF-β signal molecules should be analyzed and interpreted at a single cell level to cover the diverse and mutable functions within the disease. 

## 5. Potential Therapeutic Strategies for Fibrosis

Therapeutic strategies that target the TGF-β pathway have been developed in fibrosis based on abundant in vitro and in vivo preclinical studies. There are multiple levels of potential targets within the TGF-β pathway: (1) pro-fibrotic ligand/receptor activity inhibition, (2) anti-fibrotic ligand/receptor activation, and (3) SMAD signaling inhibition. TGFβR interacting proteins, co-activators/co-repressors, and epigenetic regulation of transcription factors have been suggested as potential targets [17]. Small molecule inhibitors, antibodies, decoy receptors, oligonucleotides, and anti-sense vectors that target TGF-β signaling factors have been proposed for therapeutic benefits [87,152,153,154]. Numerous relevant preclinical studies have associated salivary gland fibrosis with TGF-β signaling, but data on therapeutic targeting are scant. We will, therefore, discuss potential therapeutic strategies in the overall spectrum of organ fibrosis. Several TGF-β pathways targeting therapeutic candidates have been developed or are under investigation, yet the majority have not achieved satisfying clinical results. 

### 5.1. Inhibition of Pro-Fibrotic Ligand and Receptor Activity

TGF-β1 and 2, but not TGF-β3, show pro-fibrotic activity. Pro-fibrotic ligand inhibitors can be used at two points of TGF-β activation, the first being latent TGF-β activators, such as αvβ6 integrin. Antibody on αvβ6 integrin reduced fibrosis in an experimental pulmonary fibrosis model and attenuated in vitro lipopolysaccharides-induced EMT [155,156]. Latent TGF-β activator expression is site-specific and localized at areas of aberrant TGF-β activation, so that activator-targeted inhibitors have been assumed to alleviate TGF-β signaling with minimal adverse effect on other tissues. However, this has not been clinically demonstrated. αvβ6 integrin antibody BG00011 (formerly STX-100) was in phase II trial in idiopathic pulmonary fibrosis, but the clinical trial was halted due to safety issues [157]. Small molecule αvβ6 inhibitor GSK3008348 has passed phase I trial [158]. 

The second target is the activated ligand itself, neutralized by antibodies or small molecule inhibitors. A small molecule TGF-β inhibitor, pirfenidone (Esbriet^®^), has been approved as an agent for idiopathic pulmonary fibrosis in several countries and is currently one of the leading anti-fibrotic drugs in the field [159]. Nevertheless, its molecular mechanism remains unclear. It has shown promising results in phase I/II trial for diabetic nephropathy [160] and in phase II trial for focal segmental glomerulosclerosis [161]. Pan-TGF-β antibody fresolimumab (GC1008) has been in phase II trial for resistant idiopathic focal segmental glomerulosclerosis [162], and phase I trial in idiopathic pulmonary fibrosis (ClinicalTrials.gov Identifier: NCT00125385) and systemic sclerosis [163]. TGF-β1 antibody metelimumab (CAT-192) has been through phase I/II trial in systemic sclerosis but lacked clinical efficacy [164]. Another TGF-β1 antibody, LY2382770, has been in phase II trial conducted for diabetic nephropathy but was ended early because of futility issues [165]. A phase III trial on TGF-β2 antibody lerdelimumab (CAT-152) for glaucoma failed to present advanced therapeutic benefits relative to placebo [166]. 

Another method to suppress ligand activation is to trap it with soluble decoy receptors. Preclinical application of soluble TGF-β receptors or receptor fragments reduced experimental pulmonary, dermal, and renal fibrosis [167,168,169]. 

The effects of TGFβR kinase inhibitors on fibrosis have been established in rodent models. Several receptor kinase inhibitors have been developed, but there are concerns for adverse off-target effects, such as provoked inflammatory response because of the comprehensive implications TGFβR has on the signaling pathway. Topical administration of peptide 144 has been in phase II trial for dermal fibrosis and systemic sclerosis (ClinicalTrials.gov Identifier: NCT00781053, NCT00574613, respectively). Oral administration of TGFβR kinase inhibitor GW788388 reduced renal fibrosis and Chagas disease-associated cardiac fibrosis [170,171]. Another TGFβR kinase inhibitor, SB-525334, decreased pulmonary fibrosis in rodents [172,173]. 

### 5.2. Activation of Anti-Fibrotic Ligand and Receptor

Anti-fibrotic growth factors TGF-β3 and BMPs have been proposed as potential therapeutic targets to downregulate TGF-β induced transcription. TGF-β3 has an opposite preclinical ability from other TGF-β isoforms in fibrotic diseases [84]. Recombinant TGF-β3 agent, avotermin (Juvista^®^) was suggested as a drug to reduce cutaneous scarring and improve wound healing, yet it did not meet primary and secondary endpoints in a phase III trial [174]. 

Recombinant BMP2, 6, 7 and BMP agonists have reversed experimental fibrosis in preclinical studies [92,94,95,96,97,175]. Nevertheless, only BMP7 agonist THR-184 has reached phase II trial [176]. Another BMP agonist, THR-123, which is specific for ALK-3, has shown anti-fibrotic potential in experimental renal fibrosis [91]. Despite the apparent preclinical evidence of anti-fibrotic effect in BMPs, successful application for therapeutic use in the clinic is yet another challenge, as we will discuss. 

### 5.3. Inhibition of the SMAD Pathway

Intracellular targets for fibrosis within the canonical SMAD pathway have been recognized but have not yet reached clinical trial. Such targeting can be established by either inhibition of R-SMAD phosphorylation or I-SMAD activation. A small molecule alkaloid, halofuginone (HT-100), prevented or reduced experimental fibrosis in the lung, liver, and skin via inhibition of SMAD3 phosphorylation [177,178,179]. Another inhibitor, SIS3, targets SMAD3 phosphorylation and can alleviate fibrosis in diabetic nephropathy [180]. SMAD7, which can regulate both the TGF-β and BMP pathways, has been actively investigated in fibrosis, though, so far, little evidence supports its potential as a therapeutic target [28,29,30]. Adenovirus vector gene transfer of SMAD7 prevented renal and pulmonary fibrosis via interference of SMAD2/3 activity [181,182].

### 5.4. Issues in Clinical Delivery of BMP

BMPs, especially BMP7, are evident anti-fibrotic growth factors, but with convoluted limitations during clinical application. Systemic administration of BMPs to gain a valid clinical efficacy level has long been a challenge, particularly due to the impracticality of maintaining a constant BMP level by continuous exogenous ligand. The major limitation is the short half-life of BMPs when located in physiologic buffers (such as blood plasma) with under 5% of the BMP dose maintained at the targeted site [183]. Moreover, BMP7 generally has a poor cellular expression level, low solubility, and amino-terminal heterogeneity, which accounts for its poor biochemical activities [184]. Aside from the fact that recombinant proteins are expensive, higher doses of exogenous BMPs to compensate for delivery loss and overcome poor clinical efficiency levels can result in more off-target effects [185], as observed in BMP use for bone regeneration and repair [183].

Various delivery systems have been suggested to address these limitations and thus improve the clinical efficacy of BMPs. Mutation-mediated structural alterations have been applied to recombinant BMP7 to improve bioactivity and delivery efficiency [184]. Novel BMP delivery systems in the form of biomaterial carriers have been primarily reported with respect to bone regeneration. Although osteogenesis by recombinant BMP2 and 7 in carriers and scaffolds were remarkable, they had major limitations in terms of uncontrollable, burst forms of protein release [186]. As in osteogenesis, achieving a consistent and continuous BMP supply is a critical issue in the treatment of fibrosis, where carrier use is harder to apply. Adeno-associated viral vector-mediated gene therapy has been reported for BMPs in osteogenesis and BMP7-regulated neuroprotection [187,188]. Adeno-associated viral vector-mediated gene transfer of BMPs for fibrosis currently lacks preclinical evidence and needs further investigation [189].

Novel mode of action is an additional approach to improve exogenous ligand delivery. Kim et al. reported a prodrug with a protein transduction domain that mimics endogenous protein processing, which, in turn, is expected to maintain consistent BMP secretion [190]. This mode of action is currently under research for use in BMP prodrugs for fibrosis. An updated study showed that BMP7 prodrugs had a five-fold longer half-life than general recombinant BMP7 when injected within the peritoneal cavity in mice [191]. Moreover, in identical doses, the BMP7 prodrug was more efficient in inhibiting rat peritoneal fibrosis. 

## 6. Conclusions

TGF-β signaling has a pivotal role in fibrosis, including salivary gland fibrosis. Current and potential therapeutic strategies suggested for fibrosis in other organs hint at future therapeutic options for salivary gland fibrosis. Preclinical studies have claimed TGF-β/BMP signaling molecules as promising anti-fibrotic drug targets in various organ fibrosis animal models, whereas a vast majority of clinical studies have not yet achieved an acceptable level of therapeutic performance. The broad distribution and pleiotropic expression of TGF-β signaling in physiological tissue and the lack of specific delivery and maintenance of anti-fibrotic BMPs are major hurdles in targeting the TGF-β pathway for fibrosis treatment. Along with the ongoing clinical trials of anti-fibrotic drugs developed to target the TGF-β pathway at different levels, novel technologies to improve clinical delivery of BMPs are being introduced to the field. The current state of therapeutic development and research in other organs gives us insights on potential therapeutic approaches and considerations for salivary gland fibrosis.

## Abbreviations

TGF-β	Transforming growth factor beta
BMP	Bone morphogenetic protein
ECM	Extracellular matrix
TGFβR	Transforming growth factor beta receptor
ALK	Activin receptor-like kinase
(R)-SMAD	Regulated-SMAD
(Co)-SMAD	Common partner-SMAD
(I)-SMAD	Inhibitory-SMAD
LAP	Latency-associated protein
SLC	Small latent complex
LTBP	Latent TGF-β binding protein
LLC	Large latent complex
EMT	Epithelial–mesenchymal transition
EndMT	Endothelial-mesenchymal transition
MMP	Matrix metallopeptidase
SS	Sjögren’s syndrome
IL	Interleukin
Th17	T helper 17
